# A new species of *Otacilia* Thorell, 1897 (Araneae, Phrurolithidae) from Yintiaoling National Nature Reserve, Chongqing, China

**DOI:** 10.3897/BDJ.12.e124006

**Published:** 2024-04-25

**Authors:** Changbin Zheng, Yannan Mu

**Affiliations:** 1 Management Center of Yintiaoling National Nature Reserve, Chongqing, China Management Center of Yintiaoling National Nature Reserve Chongqing China; 2 Key Laboratory of Eco-environments in Three Gorges Reservoir Region (Ministry of Education), School of Life Sciences, Southwest University, Chongqing, China Key Laboratory of Eco-environments in Three Gorges Reservoir Region (Ministry of Education), School of Life Sciences, Southwest University Chongqing China

**Keywords:** Dionycha, morphology, new species, taxonomy

## Abstract

**Background:**

Phrurolithidae is a family of spiders with 395 species belonging to 26 genera distributed worldwide, of which 205 species belong to 17 genera was recorded in China.

**New information:**

A new species of the genus *Otacilia* Thorell, 1897 is described from Yintiaoling National Nature Reserve, Chongqing, China. Diagnosis, morphological description, photos of the habitus and genitalia of the new species are provided.

## Introduction

*Otacilia* Thorell, 1897, the largest genus of family Phrurolithidae, contains 137 species and is distributed in East Asia and Southeast Asia; amongst them, 114 species were reported in China (World Spider Catalog 2024). The species and studies of *Otacilia* have accelerated considerably during past decade (Liu et al. 2020, Liu et al. 2022, Mu and Zhang 2021, Mu et al. 2022, Mu and Zhang 2023). Some revisionary works in recent years have reduced the complexity of *Otacilia* by assigning species to newly-established genera (Liu et al. 2020, Zamani and Marusik 2020, Kamura 2021, Liu et al. 2022, Mu and Zhang 2022, Mu and Zhang 2023), which greatly promoted the study of *Otacilia*. While examining specimens collected from Yintiaoling National Nature Reserve, one new *Otacilia* species has been discovered and is described here: *Otaciliawuxi* sp. nov.

## Materials and methods

The specimen was preserved in 75% ethanol and was examined, illustrated, photographed and measured using a Leica M205A stereomicroscope, equipped with a drawing tube, a Leica DFC450 Camera and LAS software (Ver. 4.6). Male pedipalp was examined and illustrated after being dissected. Eye sizes were measured as the maximum dorsal diameter. Leg measurements are shown as: total length (femur, patella and tibia, metatarsus, tarsus). All measurements are in millimetres. Specimens examined here are deposited in the Collection of Spiders, School of Life Sciences, Southwest University, Chongqing, China (SWUC).

Abbreviations used in the text: ALE–anterior lateral eye; AME–anterior median eye; PLE–posterior lateral eye; PME–posterior median eye; MOA–median ocular area; pv–proventral; rv–retroventral.

## Taxon treatments

### Otacilia wuxi

Zheng & Mu sp. nov.

E3A2D256-5536-52CE-AD79-EB122549A9B1

D221A70C-FA5A-4213-94B7-0833CCB70776

#### Materials

- **Type status:** Holotype. **Occurrence:** individualCount: 1; sex: male; lifeStage: adult; occurrenceID: 73DFCC07-5E15-5A57-93EB-2E7BD54AEB5E; **Taxon:** scientificName: *Otaciliawuxi*; order: Araneae; family: Phrurolithidae; genus: Otacilia; **Location:** country: China; stateProvince: Chongqing; county: Wuxi; locality: Yintiaoling National Nature Reserve, Baiguo forest farm, Qinglong pool; verbatimElevation: 1155; verbatimLatitude: 31°30′49.88″N; verbatimLongitude: 109°49′23.60″E; **Event:** year: 2022; month: 9; day: 2; **Record Level:** institutionID: the Collection of Spiders, Southwest University; institutionCode: SWUC

#### Description

**Figure 1.**
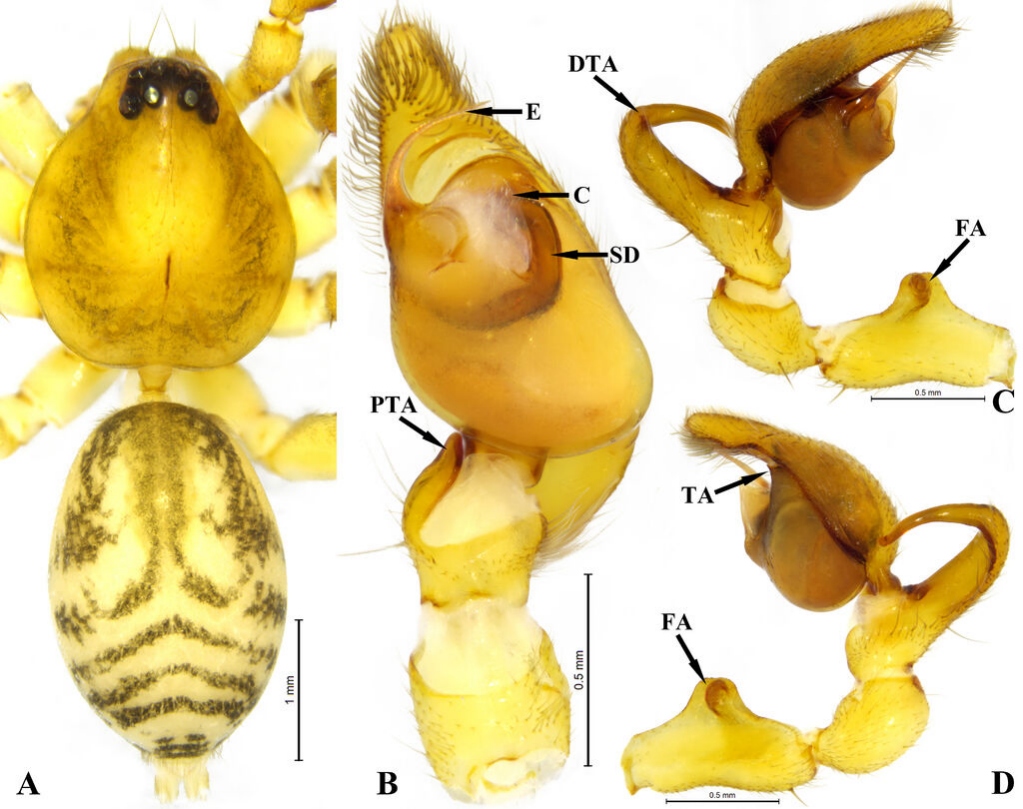
*Otaciliawuxi* sp. nov., male, holotype. **A** habitus; **B** left palp, ventral view; **C** same, prolateral view; **D** same, retrolateral view. Abbreviations: C—conductor; DTA—dorsal tibial apophysis; E—embolus; FA—femoral apophysis; PTA—prolateral tibial apophysis; SD—sperm duct; TA—tegular apophysis.

Male: total length 5.01, carapace 2.19 long, 1.91 wide; abdomen 2.59 long, 1.67 wide. Eye sizes and interdistances: AME 0.14, ALE 0.15, PME 0.11, PLE 0.13, AME–AME 0.04, AME–ALE 0.02, PME–PME 0.16, PME–PLE 0.09, ALE–PLE 0.15. MOA 0.37 long, anterior width 0.30, posterior width 0.42. Clypeal height 0.19. Chelicerae with three promarginal and eight retromarginal teeth. Measurements of legs: Ⅰ 8.86 (2.34+3.44+2.00+1.08), Ⅱ 7.05 (1.87+2.59+1.55+1.04), Ⅲ 6.18 (1.70+1.93+1.58+0.97), Ⅳ 9.46 (2.52+2.95+2.62+1.37). Spination: tibia Ⅰ pv 8 rv 8, tibia Ⅱ pv 7 rv 7, metatarsus Ⅰ pv 4 rv 4, metatarsus Ⅱ pv 3 rv 3. Legs yellow. Carapace yellow, with several indistinct shapes resembling flowing water droplets beside fovea. Abdomen yellow, with a small, thin dorsal scutum and irregular black pattern anterior and four black chevron stripes posterior (Fig. 1A).

**Palp**. Femoral apophysis high, located at middle part of femur, well-developed (Fig. 1C and D). Dorsal tibial apophysis long and large, strongly curved as semi-elliptic, base wide, tapering from middle to tip (Fig. 1C and D); prolateral tibial apophysis distinct (Fig. 1B). Tegulum bean-shaped, wider than cymbium; tegular apophysis semicircular. Conductor membranous (Fig. 1B). Sperm duct obvious, tapering from retrolateral of tegulum to embolus. Embolus long, needle-like, strongly curved retrolaterally from basal part (Fig. 1B).

Female: unknown.

#### Diagnosis

This new species resembles *O.shaoyao* Mu & Zhang, 2023 in having a similar shaped embolus and tegular apophysis, but can be recognised by: 1) the absent retrolateral tibial apophysis (vs. palmate-shaped, cf. Fig. 1B and fig. 47 in Mu and Zhang (2023)), 2) the long and semi-elliptic dorsal tibial apophysis (vs. absence, cf. Figs. 1C and D and fig. 47 in Mu and Zhang (2023)).

#### Etymology

The specific name is derived from the type locality; noun.

#### Distribution

Known only from the type locality (China: Chongqing).

#### Remarks

The specimen of this new species was collected by a Malaise trap built in the forest; however, nothing was found through sifting leaf litter. This new species is similar to *O.ailan* by lacking retrolateral tibial apophysis (Mu and Zhang 2023). However, this new species has great differences with *O.ailan*, such as the long embolus and dorsal tibial apophysis (both short in *O.ailan*), the absence of cymbium apophysis (presence in *O.ailan*), the ventral tibial protuberance indistinct (distinct ventral tibial apophysis) and the well-developed femoral apophysis (punctiform in *O.ailan*).

## Supplementary Material

XML Treatment for Otacilia wuxi
